# Supplementary material for: Nanopore sequencing of long ribosomal DNA amplicons enables portable and simple biodiversity assessments with high phylogenetic resolution across broad taxonomic scale
Source: Gigascience. 2019 Mar 2;8(5):giz006. doi: 10.1093/gigascience/giz006 (PMC6503943; doi:10.1093/gigascience/giz006)
Supplement: Response_to_Reviewer_Comments_Original_Submission.pdf [file giz006_response_to_reviewer_comments_original_submission.pdf]

Author's Response To Reviewer Comments

Close

Dear Editors
We thank the two reviewers for their constructive and helpful comments. We have now incorporated their suggested changes to our manuscript. We have provided additional detail to the introduction, methods, results and discussion and added two Supplementary Figures to better illustrate our findings.
We hope that the manuscript will now be deemed acceptable for publication in Gigascience.
Sincerely,
Henrik Krehenwinkel

Reviewer reports:
Reviewer #1: The authors present us an rDNA-based barcoding and phylogeny study using a MinION sequencing platform. It is an instructive trial and I suggest the editor make it published after addressing several issues as follows:

1. The authors should be cautious of scientific writing and provide evidences to what you have written. For example, the authors stated that one of the pitfalls of mitochondrial genes is the risk of homoplasy of divergent lineages because of saturation. However, a short standard COXI barcode of length ca. 600 bp can hold a variety of 4^600, 4^200 even only take into the third position into account, which is far more than the species number on earth. In addition, nowadays mitochondrial genes are well known of its limitation in phylogeny works due to reasons mentioned by the authors in lines 80-90, but I image that most of these limitations should affect much on demographic history inferences for single species or phylogenetic work of closely related species, rather than biodiversity oriented and alpha or beta diversity based ecological works. I encourage the authors to pay more attentions on their writing to avoid biased texts which may mislead readers.
- We fully acknowledge the almost unlimited number of informative sites in a COI barcode. COI is certainly well suited to distinguish species and this is not affected by homoplasy. We simply meant that its utility to resolve phylogenetic divergence is limited by homoplastic sites. At deep phylogenetic divergence, the sequence saturates with mutations, making it hard to properly reconstruct relationships. We have made this clearer in the introduction.

2. Same to 1, at line 116, in opposite to what the authors stated, ITS2 is proposed to be the optimal barcode marker for plants and fungi.
- We personally have found considerable drop out of arthropod specimens during PCR using common universal ITS primers, but agree that it is a widely used and well-suited taxonomic marker for many other lineages. We have rephrased the according section. We now particularly focus on the difficulty of aligning the extremely variable ITS sequences across divergent lineages.

3. Although the authors mentioned the Pacbio sequencer as an alternative method to explore community compositions in lines 123-127, I think it needs more words to make it clear that the CCS (circular consensus sequencing) tech of Pacbio sequencing platform may be more suitable for amplicons-based barcoding and biodiversity work. However, comparing to Nanopore tech, it can hardly be conducted in a real-time way and in the field.
- We have rewritten the relevant sections. We highlight the utility and advantage of the CSS sequencing. We then focus on the advantage of the MinION of being a portable and easily accessible device.

4. I agree that an empirical experiment is necessary to test how Nanopore tech works on the estimation of metazoan community diversity. However, what impedes MinION from amplicons-based diversity study is its lower per base accuracy. The authors should understand that the alpha diversity inflation is still one of the major concerns even using the widely applied HiSeq sequencing platform which holds much higher sequencing accuracy. I believe the MinION-based study, at current stage, is far from being worry about such problems. I am afraid that researchers in this field are still skeptical of its applicability in metabarcoding at current stage. As I see in the authors' work, you manually mixed phylogenetically divergent species - species from different orders - to avoid taxonomic assignment issues. But the authors should also be aware that such a design has less practical guiding significances.
- We agree that the MinION is not yet ready for routine community analysis, as also shown by our data. Expecting difficulties with this system, we have used highly simplified community samples, to explore its potential utility for community analysis. We acknowledge that our mock communities are not directly comparable to natural communities and have revised the methods and discussion to highlight this. Finding highly biased results in these simplified communities already highlights the possible difficulties of this system in real communities.

5. For the consensus sequences of plants or fungi mentioned in lines 408 - 410, if they are food chain derived, have you ever tried to cluster reads at first, then call consensus for each cluster? Or as you mentioned in lines 650 -652, check taxonomic composition by blasting a reference library before assembly.
- We have tried this and found that for these samples, the majority or even all assigned sequences belonged to the non-targeted species; the host was almost undetectable in these cases. For example, we did not find a single Zophobas beetle sequence in an extract of Zophobas larvae, but highly abundant rye DNA sequences. We have added an explanatory sentence to the results.

6. The authors mentioned that coverage larger than 300 can lead to a decrease of consensus accuracy. It deserves further scrutiny to get reasonable explanations. In addition, read number increased a lot per sample with a minibar setting of edit distance of 4, which, however, generated less accurate consensuses. Are there any correlations between these two observations?
- This difference is visible in the plot, but it is not significant. We have added this information in the results. It is also visible that there is an overlap of consensus accuracy at coverages > 300 and < 300. Hence, only part of the samples showed a lower consensus accuracy at high coverage. We assume that this is due to some samples randomly getting assigned more wrongly

demultiplexed samples at high coverages. There always seems to be a small carryover between indexes. These wrongly assigned sequences may affect consensus building. At an edit distance of four, we indeed found a considerable increase of wrongly assigned sequences, e.g. cross contamination between samples. This affected consensus building and led to inaccurate consensus sequences. We have added this information to the results.

7. How do you annotate the rDNA to separate the different segments - 18S, 5.8S, ITS, et al.
- We used annotated reference sequences from Genbank. We have now included this information in methods

8. Is there any data that support what you mentioned in lines 661 - 662: "indices of 20 or 30 bp attached to primers doesn't strongly affect CPR efficiency"?
It is common practice to use tailed primers in Illumina amplicon sequencing, which are longer than 50 bp and work efficiently. Our indexes are considerably shorter. Yet, on the other hand, the targeted amplicon is also much longer. To our knowledge, there is no proof for our assumption. We have thus rephrased the according sentence in the discussion and removed the statement
-
9. Please make sure correct citations, e.g. I don't think reference number 48 talked about anything related to what you stated there at line 666.
- The reference was corrected

10. Others:

Supplemental figure 1. Please add the unite of your Y axis, should be in percent, isn't it?
- The unit added to the figure is percent

Line 255, is it minimap2?
- We have used minimap, not minimap2. We have, however recently tested minimap2 and it did not yield better results.

Line 285, do you mean crossover?
- We mean samples being wrongly assigned due to indexes being misidentified due to sequencing error. We have reworded this to crossover.

Reviewer #2: The manuscript entitled 'Nanopore sequencing of long ribosomal DNA amplicons enables portable and simple biodiversity assessments with high phylogenetic resolution across broad taxonomic scale' by Krehenwinkel and collaborators aims to evaluate the use of Nanopore to produce high quality consensus sequences for a long fragment spanning the rDNA region. The authors evaluate the usage of the different genes and two gene interspace regions contained within the amplified fragment and that present different levels of nucleotide variability, for a comparative analysis at different taxonomic levels, most notably within arthropods and specifically within a group of spiders.

The manuscript is well written, and mostly clear in the ideas. The methods and experiments presented seem to complement each other and are ultimately showing the potentiality of using the methodology described in the manuscript for barcoding a long and highly informative region of the rDNA 'operon' for any given arthropod (or potentially eukaryote) species in the location where it is sampled, independently of the local laboratory infrastructure. The use of such 'portable approaches' for the study of biodiversity are highly desirable in times in which biodiversity is fast declining and samples exporting from regions representing biodiversity hotspots are facing more severe regulations. Certainly, performing methods locally but with infrastructure that can be easily transported from abroad if needed is a great advantage.

My main criticism to the text is the confusion made between biases produced by long and short-read technologies and those produced by the different types of amplicons generated. The authors should differentiate between PCR efficiency and sequencing technologies across the paper. For example, instead of 'long-read metabarcoding' please call it 'long-amplicon metabarcoding'. This makes it clear that the problems found are due to the PCR, potentially due to its long-range nature. However, it should also be made clear that optimization of PCR conditions is needed for both short and long-range when new primers are developed. Note that even though it is a natural expectation that long reads will be used for sequencing long amplicons and short reads for short amplicons, this is not a rule. Illumina can be used for shotgun sequencing of long amplicons and Nanopore could potentially be used for short amplicons or even concatenated short amplicons.
Abstract

I suggest to change in line 48 long-read by long-amplicon or by 'long-amplicon approaches combined with long-sequencing technologies'.
- Was done throughout

Background

Line 81 - the authors are mostly talking about the COI gene and not mitochondrial DNA in general. Mitogenomes also have a combination of genes with more or less expected levels of divergence between species. Some genes, such as the non-coding 16S and 12S have very conserved regions across taxa. If one could potentially amplify different mitochondrial genes across taxa in one single amplicon, the power would be probably at least similar to the rDNA operon, but apart from the issues already described by the authors regarding the peculiarities of the mitogenome such as maternal inheritance and the possibility for introgressive hybridization, mitogenomes might vary a lot in synteny, content and number of gene copies in some phyla (e.g. Fungi) and are therefore not exactly useful for amplifying a number of homologous regions consistently across eukaryotes.
- We have also considered using mitochondrial DNA, which has many advantages as well. 16S and 12S do indeed have fairly conserved sequence stretches allowing the design of primers, which efficiently amplify a wide range of taxa. However, they are not nearly as conserved as nuclear rDNA. In our experience, universal 12S or 16S primers may allow us to amplify all taxa across an order or phylum, but not a whole domain as nuclear rDNA does.
In the long run, we aim to develop a combined approach utilizing nuclear and mitochondrial

long amplicon information. We have added additional information on this in the discussion.

Having said that, I never looked in more detailed into this possibility, so there might be certain genes that always occur in synteny in mitogenomes. But I agree that mitochondrial DNA is not always representative of phylogenies. This brings us to the general questions that should be posted after line 111. Are the peculiarities of the rDNA operon a potential bias for some phylogenetic inferences? For example, the variable (and unknown) number of copies across species that may or may not be all identical. I would appreciate some acknowledgement of the potential uncertainties on phylogenies based on rDNA already in the introduction.
- We now acknowledge the limitations of single rDNA sequences in the introduction and discussion. E.g. nuclear rDNA can also be prone to paralogs and possibly pseudogenization. We also highlight the combination of long mitochondrial and nuclear amplicons as an ideal solution for future barcoding applications in the discussion.

Line 116 - it is true the ITS regions are too variable for designing universal primers, but they are flanked by conserved regions, and to the best of my knowledge ITS2 is not as variable in length as ITS1. So, instead of describing the variability of ITS regions as impeditive to short-amplicon primers design, I would rather discuss the fact that it cannot be aligned among unrelated taxa, and are not suitable for deeper phylogenies. Besides, it can only be used for taxonomical assignment if a somehow related group is represented in the database.
- We acknowledge this and have rewritten the according section in the introduction.

Line 133 - I would add consensus sequences 'from single individuals'. I was confused at first thinking that Nanopore could maybe do some sort of 'circular consensus', but if the consensus sequences are produced by homologous sequences from a single individual this should be made clear.
- Has been made clear in the introduction

Line 141 - I would rephrase 'universal eukaryote'. Even though the primers could potentially work for all eukaryotes, there was no representative collection tested, and the authors stated themselves that there was a focus in animals.
- Was rephrased

Data Description and Analyses

Line 201 - following the idea above of exploring the universality of the primers, I would like to see some sort of figure or graph showing the representativeness of the different groups of eukaryotes in the 1000 sequences used for the primers design.
- We have added this graph as a supplementary figure

Line 214 - How was the quantification on an agarose gel performed? I would suggest a description how this was done and an evaluation of the pooling method in the Results/Discussion as fluctuations on samples sequence numbers may highly influence the

efficiency and costs of the method.
- We have added the details for this approach in the methodology. We acknowledge that it may introduce some biases and have added a discussion for this

Line 221 - Please inform the concentration of AMPure beads utilized
- We used 0.75 X beads on 100 ul, e.g. 75 ul of beads. The volume was added to the manuscript

Results

Line 383 and Fig.2 - the authors state that at a distance of 4, samples had an increase in wrongly assigned sequences and a significantly lower accuracy in the consensus generated. However, what is shown in Fig. 2 is a box plot of pairwise distances of Nanopore sequences assigned to the sample against the Illumina consensus. How do the authors know that the sequences were wrongly assigned? Could they be assigned to other samples based on sequence distance? Is there a real change in the consensus sequence generated by the sequences assigned to a sample at a distance of 4? If so, why is that? Due to more indels and/or more mismatches? What are the features of the newly assigned sequences that decrease the accuracy of the consensus? Could the higher distance at the barcode also incorporate sequences with more errors (i.e. is the number of errors in barcodes correlated to lower quality/more errors)? Are the errors distributed throughout the sequences? In my view it's important
to understand the causes of lower accuracy, because absolute numbers, such as 2, 3 or 4 mismatches, might not represent the same issues when different barcode length, sequences or combinations are used.
- We have blasted the raw reads to explore potential carry over between indexes. At an edit distance of four, we indeed found a considerable increase of wrongly assigned sequences, e.g. cross contamination between samples. This affected consensus building and led to inaccurate consensus sequences. We have added this information to the results.

Line 420 - please show examples of alignments with errors clustered in indel regions in the supplementary material. It is important for the reader to understand the patterns of errors found.
- We added a supplementary figure detailing the increased error at homopolymers.

Line 429 - what could be the reason for a decrease in accuracy in higher coverages? Is this increase stochastic and no significant? Or is the incorporation of sequences with more (and maybe slightly repetitive) errors causing differences in the consensus? It would be very interesting to understand if the consensus creation is very sensitive to accumulation of identical errors, even if in small rates.
- As stated above, this difference was not significant. We have added this information in the results. It is also visible that there is an overlap of consensus accuracy at coverages > 300 and < 300. Hence, only part of the samples showed a lower consensus accuracy at high coverage. We assume that this is due to some samples randomly getting assigned more wrongly demultiplexed samples at high coverages. There always seems to be a small carryover between indexes. These

wrongly assigned sequences may affect consensus building.

Lines 431 to 449 and Suppl. Figures 3 and 4 - even though I understand the value of presenting and summarizing results, the authors should not treat the data as representative neither for animals nor for plants. Please refer to the main groups analyzed (Arachnids, Insects and Magnoliopsida) and if there is an interest to compare to animals and plants in general, pick representative sequences from both groups (animals and plants) from public databases and present a comparison. For the data presented here, my suggestion would be one single boxplot graph for length difference presenting Arachnids, Insects and if wanted Magnoliopsida including both lengths excluding and including ITS regions, for a better understanding of the differences between full versus coding-only lengths. Another graph (or figure number) can summarize the same way the GC content.
- We have remade the plots as suggested by the reviewer. And have rewritten the according text section in the results.

Figures 3, 4 and 5 are presented before they are mentioned in the text.
- Has been corrected

Lines 471 to 483 and figure 3 - I wonder here what the value is in building such a phylogenetic tree including non-representative but yet arbitrarily picked species from three different kingdoms. Is the intent to show that the sequences produced by Nanopore are as accurate as sequences produced by other technologies and that differences/errors do not affect phylogenetic reconstruction? In that case, I would compare the tree with the data from same/similar species from databases for each group. Or is the intent showing that rDNA can be used to reconstruct true phylogenies for the groups? This doesn't seem to be part of the goals, but would demand other tests, again including many more sequences from databases. If the authors are presenting novelties and would like to place some group phylogenetically, there is nothing wrong (or better, it's the right thing to go) in picking representative sequences from databases for showing the correct phylogenetic placement of a group.
- We aimed to show a widely applicable method here, which is why we used different other taxa besides arthropods, even though our focus is clearly on arthropods. We did not aim to reconstruct the tree of life for these groups, but merely show that it is possible to amplify, sequence and align rDNA sequences across different domains of the eukaryote tree of life. Our primary goal is to allow amplification of all organisms from a given biological community; importantly, this provides a means to generate metrics of similarity between communities based on quantitative phylogenetic data. If Figure 3 is not important, we are happy to move it to supplement. Our sampling is particularly focused on arthropods, for which we present a wide range of taxa. Starting at the phylum level, we move into the order spiders and show that the recovered phylogeny is well comparable to recent work based on whole transcriptomes. We then even move to the genus level and present a detailed analysis in a genus of Hawaiian spiders. Reconstructing the tree of life with additional database sequences for all eukaryote groups would extend beyond the scope of our study, which already is extensive and has multiple facets.

Lines 485 to 499 - Spiders are surely much better represented than other groups. But if there are other sequences in databases not represented here, I would include them. The question always goes back to the intent. Is it to show that the authors are contributing with valuable and correct rDNA sequences to populate databases? Then the improvement in phylogeny reconstruction should involve all sequences available and for which taxonomy can be trusted (I'm accounting here for possible errors or uncertainties in databases). This would reinforce the value of the approach in creating new references for an important and informative marker (or better saying, cluster of markers with different levels of divergence among different taxonomical groups), the rDNA region.
- Whole rDNA clusters are still not very well represented in public databases. This holds particularly true for spiders, for which very few whole rDNA sequences are present in the databases. It was not our aim to reconstruct a complete spider tree of life, but rather to show that the rDNA cluster allows to recover the known phylogenetic divergence for the limited set of taxa we have used here. Our data already covers a considerable portion of the araneaomorph spider tree of life and we present the resolution of rDNA sequence across multiple taxonomic levels, from family down to species. The spider tree of life is well resolved by RNA seq data. We simply show that the rDNA cluster alone resolves a very congruent phylogeny at multiple levels.

Line 531 and 532, Fig.6 and Discussion - the overlap between inter and intra-specific distance in rDNA might seem small but could have serious consequences if not interpreted well. Please show if in the dataset the distance would be impeditive of taxonomical assignment based on distance for some lineages. One simple way would be to highlight the circles (e.g. make them darker) in Fig. 6 and suppl. Fig 5 with high intra-specific variability in rDNA for both intra and inter-specific distances. If the highlighted circles have high distance for inter-specific comparisons as well, this would not affect taxonomical classification, at least for those set of species/specimens presented.
- The overlap between distances for the rDNA cluster is caused by only very few species. In fact, it is only a single case of high intraspecific distance basing the overlap. This case possibly involves a cryptic species pair. Which also shows a high distance in COI. We are currently examining material of the species morphologically, to explore this further. Also, the species shows a considerably higher interspecific distance to other Tetragnatha species, than its intraspecific distance. A pair of very closely related species from Maui show the lowest interspecific distance. We have added some more details on this in the results section

I wonder if the inter X intra-specific distances gap in COI is a natural one, or if in some cases COI was itself used for re-defining species boundaries, which would make the analysis redundant. In any case, it seems that in general it could be a good approach, even if it increases the level of
Complexity, to sequence both rDNA and COI, as mentioned in the Discussion. I just wonder that, if COI could also be sequenced in the field with a similar approach (i.e. using Nanopore), as some samples might never reach the lab in the original country where the research is being conducted.
- The observed barcode gap in COI is in fact natural. We have now explained this in the text. All species, which we used for this study were identified morphologically before we performed

barcoding analysis. Also, we are currently exploring the possibility of using a combined approach of long mitochondrial and nuclear rDNA amplicons for taxonomic analyses, which we believe would be an ideal solution.

Lines 569-586 - The Illumina metabarcoding seem highly accurate when compared to Nanopore in Suppl. Fig 8 (please correct in line 574 the figure number), but this does not confirm that Illumina is quantitatively accurate, as the long amplicons generated huge biases. In fact, some taxa seem to have more than 2-fold difference in the Illumina results compared to their original frequency in the mock community based on Fig. 7. Was the adjustment per taxon performed as in reference [34]?
- Illumina sequencing was not perfectly accurate quantitatively and we did not correct read abundances. However, Illumina sequences recovered abundance trends very well. We have toned down the relevant sections in the results and discussion and added additional explanations. However, the bias observed by MinION sequencing was considerably higher than that found by Illumina.

Discussion
Line 616 - I would like to see a bit deeper discussion on the feasibility for developing universal primers for sequencing full or partial mitogenomes across taxa, especially considering gene synteny and content within eukaryotes, apart from nucleotide variation.
- We have added this in the discussion. We fully agree that adding long mitochondrial amplicons would be a great complement to our work. Ideally, one would rely on both markers. We have added additional details in the discussion highlighting the need for multi locus approaches and a combination of mitochondrial and nuclear data.

Lines 625-626 - please rephrase to something similar to: long rDNA amplicons can potentially be amplified across diverse eukaryote taxa, here largely demonstrated in arthropods and arachnids, and in very small scale in fungi and plants.
- Was rephrased

Line 654 - rDNA is not only diploid but present in multiple (and unknown) number of copies, that might not be identical
- We have added additional sentences in the introduction and discussion, considering the possible problem with multi copy rDNA markers.

Line 661 - it is not clear that even longer tails would not affect PCR, please either add a reference to confirm the statement or change it.
- As stated above, long primer tails are commonly used for Illumina sequencing. But to our knowledge their effect was not exhaustively tested yet. Also, our amplicon is much longer than typical Illumina sequenced amplicons. As we did not test it, we have removed the statement.

Line 666 - reference number is wrong, maybe [47]?
- Was corrected

Nanopore metabarcoding - I think explanations are quite reasonable for justifying why certain taxonomical groups, especially those with shorter rDNA regions, would be preferentially amplified. However, given that the whole study focused on spiders, could the conditions be better optimized for spiders rather than other arthropods?
- The protocol could probably work better for groups like spiders, which show small variation in amplicon length. We are currently testing and optimizing our protocol in that regard. We have added additional explanations and details in the methods and discussion.
References

[22] is now published in Mol Ecol Resources
- Was changed

Close
