# Supplementary material for: Nanopore sequencing of long ribosomal DNA amplicons enables portable and simple biodiversity assessments with high phylogenetic resolution across broad taxonomic scale
Source: Gigascience. 2019 Mar 2;8(5):giz006. doi: 10.1093/gigascience/giz006 (PMC6503943; doi:10.1093/gigascience/giz006)
Supplement: Response_to_Reviewer_Comments_Revision_1.pdf [file giz006_response_to_reviewer_comments_revision_1.pdf]

Author's Response To Reviewer Comments

Close

Dear Dr. Edmunds,
thank you and the two reviewers for your effort in improving our manuscript. We have addressed the few additional changes raised by Reviewer 1 and hope the manuscript is now deemed acceptable for publication.
Sincerely,
Henrik Krehenwinkel

Reviewer reports:
Reviewer #1: Most of the issues have been well addressed except for the ones regrading to mutation saturations of COI gene and the biased description of ITS regions.

The authors stated that "The phylogenetic resolution offered by short barcodes is very limited, as they contain only a restricted number of informative sites. This problem is exacerbated by the fast evolutionary rate of mitochondrial DNA, which leads to a quick saturation with mutations, increasing the probability of homoplasy." For DNA sequences, homoplasy can hardly be avoided due to its four-state nature. However, as I mentioned before, COI gene has > 600 sites, it is going to be extremely rare or, I would say, impossible for the entire gene getting saturated. The authors may want to provide citations here to illustrate how mutation saturation affect phylogenetic resolution?

• We do not question the utility of COI as a barcode marker, for which it is very well suited. Our main intention was to highlight the utility of backing up mitochondrial information with nuclear data. The sentence in question is really not essential to communicate that point. We thus deleted the according sentence.

For the ITS, as another reviewer also mentioned, ITS2 region is widely utilized to serve as barcode sequences for fungi and plants and less variable in length than other ITS regions. The authors may want to add an unbiased description of ITS regions in their main text.

• We have now added some additional detail on the differences between ITS1 and ITS2.

Reviewer #2: The authors of the manuscript entitled 'Nanopore sequencing of long ribosomal DNA amplicons enables portable and simple biodiversity assessments with high phylogenetic resolution across broad taxonomic scale' have addressed all my concerns and suggestions and I am happy to recommend this manuscript for publication.

I would recommend to move Figure 3 to supplementary material, as this is merely a confirmation that the sequences do not produce false phylogenetic signal.

• We feel this figure is important, as it nicely highlight the broad taxonomic utility of our method. We would thus prefer to leave it in the main text.

Close
