# Supplementary material for: Nanopore sequencing of long ribosomal DNA amplicons enables portable and simple biodiversity assessments with high phylogenetic resolution across broad taxonomic scale
Source: Gigascience. 2019 Mar 2;8(5):giz006. doi: 10.1093/gigascience/giz006 (PMC6503943; doi:10.1093/gigascience/giz006)
Supplement: Reviewer_1_Report_Original_Submission -- Shanlin Liu — 9/5/2018 Reviewed [file giz006_reviewer_1_report_original_submission.pdf]

Reviewer Report

Title: Nanopore sequencing of long ribosomal DNA amplicons enables portable and simple biodiversity assessments with high phylogenetic resolution across broad taxonomic scale

Version: Original Submission **Date:** 9/5/2018

Reviewer name: Shanlin Liu

Reviewer Comments to Author:

The authors present us an rDNA-based barcoding and phylogeny study using a MinION sequencing platform. It is an instructive trial and I suggest the editor make it published after addressing several issues as follows:
1. The authors should be cautious of scientific writing and provide evidences to what you have written. For example, the authors stated that one of the pitfalls of mitochondrial genes is the risk of homoplasy of divergent lineages because of saturation. However, a short standard COXI barcode of length ca. 600 bp can hold a variety of 4^600, 4^200 even only take into the third position into account, which is far more than the species number on earth. In addition, nowadays mitochondrial genes are well known of its limitation in phylogeny works due to reasons mentioned by the authors in lines 80-90, but I image that most of these limitations should affect much on demographic history inferences for single species or phylogenetic work of closely related species, rather than biodiversity oriented and alpha or beta diversity based ecological works. I encourage the authors to pay more attentions on their writing to avoid biased texts which may mislead readers.
2. Same to 1, at line 116, in opposite to what the authors stated, ITS2 is proposed to be the optimal barcode marker for plants and fungi.
3. Although the authors mentioned the Pacbio sequencer as an alternative method to explore community compositions in lines 123-127, I think it needs more words to make it clear that the CCS (circular consensus sequencing) tech of Pacbio sequencing platform may be more suitable for amplicons-based barcoding and biodiversity work. However, comparing to Nanopore tech, it can hardly be conducted in a real-time way and in the field.
4. I agree that an empirical experiment is necessary to test how Nanopore tech works on the estimation of metazoan community diversity. However, what impedes MinION from amplicons-based diversity study is its lower per base accuracy. The authors should understand that the alpha diversity inflation is still one of the major concerns even using the widely applied HiSeq sequencing platform which holds much higher sequencing accuracy. I believe the MinION-based study, at current stage, is far from being worry about such problems. I am afraid that researchers in this field are still skeptical of its applicability in metabarcoding at current stage. As I see in the authors' work, you manually mixed phylogenetically divergent species - species from different orders - to avoid taxonomic assignment issues. But the authors should also be aware that such a design has less practical guiding significances.
5. For the consensus sequences of plants or fungi mentioned in lines 408 - 410, if they are food chain derived, have you ever tried to cluster reads at first, then call consensus for each cluster? Or as you mentioned in lines 650 -652, check taxonomic composition by blasting a reference library before

assembly.
6. The authors mentioned that coverage larger than 300 can lead to a decrease of consensus accuracy. It deserves further scrutiny to get reasonable explanations. In addition, read number increased a lot per sample with a minibar setting of edit distance of 4, which, however, generated less accurate consensuses. Are there any correlations between these two observations?
7. How do you annotate the rDNA to separate the different segments - 18S, 5.8S, ITS, et al.
8. Is there any data that support what you mentioned in lines 661 - 662: "indices of 20 or 30 bp attached to primers doesn't strongly affect CPR efficiency" ?
9. Please make sure correct citations, e.g. I don't think reference number 48 talked about anything related to what you stated there at line 666.
10. Others:
Supplemental figure 1. Please add the unite of your Y axis, should be in percent, isn't it?
Line 255, is it minimap2?
Line 285, do you mean crossover?

Methods

Are the methods appropriate to the aims of the study, are they well described, and are necessary controls included? Choose an item.

Conclusions

Are the conclusions adequately supported by the data shown? Choose an item.

Reporting Standards

Does the manuscript adhere to the journal's guidelines on minimum standards of reporting? Choose an item.

Choose an item.

Statistics

Are you able to assess all statistics in the manuscript, including the appropriateness of statistical tests used? Choose an item.

Quality of Written English

Please indicate the quality of language in the manuscript: Choose an item.

Declaration of Competing Interests

Please complete a declaration of competing interests, considering the following questions:

- Have you in the past five years received reimbursements, fees, funding, or salary from an organisation that may in any way gain or lose financially from the publication of this manuscript, either now or in the future?
- Do you hold any stocks or shares in an organisation that may in any way gain or lose financially from the publication of this manuscript, either now or in the future?
- Do you hold or are you currently applying for any patents relating to the content of the manuscript?
- Have you received reimbursements, fees, funding, or salary from an organization that holds or has applied for patents relating to the content of the manuscript?
- Do you have any other financial competing interests?
- Do you have any non-financial competing interests in relation to this paper?

If you can answer no to all of the above, write 'I declare that I have no competing interests' below. If your reply is yes to any, please give details below.

I declare that I have no competing interests

I agree to the open peer review policy of the journal. I understand that my name will be included on my report to the authors and, if the manuscript is accepted for publication, my named report including any attachments I upload will be posted on the website along with the authors' responses. I agree for my report to be made available under an Open Access Creative Commons CC-BY license (http://creativecommons.org/licenses/by/4.0/). I understand that any comments which I do not wish to be included in my named report can be included as confidential comments to the editors, which will not be published.

I agree to the open peer review policy of the journal

To further support our reviewers, we have joined with Publons, where you can gain additional credit to further highlight your hard work (see: https://publons.com/journal/530/gigascience). On publication of this paper, your review will be automatically added to Publons, you can then choose whether or not to claim your Publons credit. I understand this statement.

Yes Choose an item.
