# Supplementary material for: Nanopore sequencing of long ribosomal DNA amplicons enables portable and simple biodiversity assessments with high phylogenetic resolution across broad taxonomic scale
Source: Gigascience. 2019 Mar 2;8(5):giz006. doi: 10.1093/gigascience/giz006 (PMC6503943; doi:10.1093/gigascience/giz006)
Supplement: Reviewer_1_Report_Revision_1 -- Shanlin Liu — 11/26/2018 Reviewed [file giz006_reviewer_1_report_revision_1.pdf]

Reviewer Report

Title: Nanopore sequencing of long ribosomal DNA amplicons enables portable and simple biodiversity assessments with high phylogenetic resolution across broad taxonomic scale

Version: Revision 1 **Date:** 11/26/2018

Reviewer name: Shanlin Liu

Reviewer Comments to Author:

Most of the issues have been well addressed except for the ones regrading to mutation saturations of COI gene and the biased description of ITS regions.
The authors stated that "The phylogenetic resolution offered by short barcodes is very limited, as they contain only a restricted number of informative sites. This problem is exacerbated by the fast evolutionary rate of mitochondrial DNA, which leads to a quick saturation with mutations, increasing the probability of homoplasy." For DNA sequences, homoplasy can hardly be avoided due to its four-state nature. However, as I mentioned before, COI gene has > 600 sites, it is going to be extremely rare or, I would say, impossible for the entire gene getting saturated. The authors may want to provide citations here to illustrate how mutation saturation affect phylogenetic resolution?
For the ITS, as another reviewer also mentioned, ITS2 region is widely utilized to serve as barcode sequences for fungi and plants and less variable in length than other ITS regions. The authors may want to add an unbiased description of ITS regions in their main text.

Methods

Are the methods appropriate to the aims of the study, are they well described, and are necessary controls included? Choose an item.

Conclusions

Are the conclusions adequately supported by the data shown? Choose an item.

Reporting Standards

Does the manuscript adhere to the journal’s guidelines on minimum standards of reporting? Choose an item.

Choose an item.

Statistics

Are you able to assess all statistics in the manuscript, including the appropriateness of statistical tests used? Choose an item.

Quality of Written English

Please indicate the quality of language in the manuscript: Choose an item.

Declaration of Competing Interests

Please complete a declaration of competing interests, considering the following questions:

- Have you in the past five years received reimbursements, fees, funding, or salary from an organisation that may in any way gain or lose financially from the publication of this manuscript, either now or in the future?
- Do you hold any stocks or shares in an organisation that may in any way gain or lose financially from the publication of this manuscript, either now or in the future?
- Do you hold or are you currently applying for any patents relating to the content of the manuscript?
- Have you received reimbursements, fees, funding, or salary from an organization that holds or has applied for patents relating to the content of the manuscript?
- Do you have any other financial competing interests?
- Do you have any non-financial competing interests in relation to this paper?

If you can answer no to all of the above, write 'I declare that I have no competing interests' below. If your reply is yes to any, please give details below.

I declare that I have no competing interests

I agree to the open peer review policy of the journal. I understand that my name will be included on my report to the authors and, if the manuscript is accepted for publication, my named report including any attachments I upload will be posted on the website along with the authors' responses. I agree for my report to be made available under an Open Access Creative Commons CC-BY license (http://creativecommons.org/licenses/by/4.0/). I understand that any comments which I do not wish to be included in my named report can be included as confidential comments to the editors, which will not be published.

I agree to the open peer review policy of the journal

To further support our reviewers, we have joined with Publons, where you can gain additional credit to further highlight your hard work (see: https://publons.com/journal/530/gigascience). On publication of this paper, your review will be automatically added to Publons, you can then choose whether or not to claim your Publons credit. I understand this statement.

Yes Choose an item.
