# Supplementary material for: Nanopore sequencing of long ribosomal DNA amplicons enables portable and simple biodiversity assessments with high phylogenetic resolution across broad taxonomic scale
Source: Gigascience. 2019 Mar 2;8(5):giz006. doi: 10.1093/gigascience/giz006 (PMC6503943; doi:10.1093/gigascience/giz006)
Supplement: Reviewer_2_Report_Original_Submission -- Camila Mazzoni — 9/20/2018 Reviewed [file giz006_reviewer_2_report_original_submission.pdf]

Reviewer Report

Title: Nanopore sequencing of long ribosomal DNA amplicons enables portable and simple biodiversity assessments with high phylogenetic resolution across broad taxonomic scale

Version: Original Submission **Date:** 9/20/2018

Reviewer name: Camila Mazzoni

Reviewer Comments to Author:

The manuscript entitled 'Nanopore sequencing of long ribosomal DNA amplicons enables portable and simple biodiversity assessments with high phylogenetic resolution across broad taxonomic scale' by Krehenwinkel and collaborators aims to evaluate the use of Nanopore to produce high quality consensus sequences for a long fragment spanning the rDNA region. The authors evaluate the usage of the different genes and two gene interspace regions contained within the amplified fragment and that present different levels of nucleotide variability, for a comparative analysis at different taxonomic levels, most notably within arthropods and specifically within a group of spiders.

The manuscript is well written, and mostly clear in the ideas. The methods and experiments presented seem to complement each other and are ultimately showing the potentiality of using the methodology described in the manuscript for barcoding a long and highly informative region of the rDNA 'operon' for any given arthropod (or potentially eukaryote) species in the location where it is sampled, independently of the local laboratory infrastructure. The use of such 'portable approaches' for the study of biodiversity are highly desirable in times in which biodiversity is fast declining and samples exporting from regions representing biodiversity hotspots are facing more severe regulations. Certainly, performing methods locally but with infrastructure that can be easily transported from abroad if needed is a great advantage.

My main criticism to the text is the confusion made between biases produced by long and short-read technologies and those produced by the different types of amplicons generated. The authors should differentiate between PCR efficiency and sequencing technologies across the paper. For example, instead of 'long-read metabarcoding' please call it 'long-amplicon metabarcoding'. This makes it clear that the problems found are due to the PCR, potentially due to its long-range nature. However, it should also be made clear that optimization of PCR conditions is needed for both short and long-range when new primers are developed. Note that even though it is a natural expectation that long reads will be used for sequencing long amplicons and short reads for short amplicons, this is not a rule. Illumina can be used for shotgun sequencing of long amplicons and Nanopore could potentially be used for short amplicons or even concatenated short amplicons.

Abstract

I suggest to change in line 48 long-read by long-amplicon or by 'long-amplicon approaches combined with long-sequencing technologies'.

Background

Line 81 - the authors are mostly talking about the COI gene and not mitochondrial DNA in general. Mitogenomes also have a combination of genes with more or less expected levels of divergence

between species. Some genes, such as the non-coding 16S and 12S have very conserved regions across taxa. If one could potentially amplify different mitochondrial genes across taxa in one single amplicon, the power would be probably at least similar to the rDNA operon, but apart from the issues already described by the authors regarding the peculiarities of the mitogenome such as maternal inheritance and the possibility for introgressive hybridization, mitogenomes might vary a lot in synteny, content and number of gene copies in some phyla (e.g. Fungi) and are therefore not exactly useful for amplifying a number of homologous regions consistently across eukaryotes. Having said that, I never looked in more detailed into this possibility, so there might be certain genes that always occur in synteny in mitogenomes. But I agree that mitochondrial DNA is not always representative of phylogenies. This brings us to the general questions that should be posted after line 111. Are the peculiarities of the rDNA operon a potential bias for some phylogenetic inferences? For example, the variable (and unknown) number of copies across species that may or may not be all identical. I would appreciate some acknowledgement of the potential uncertainties on phylogenies based on rDNA already in the introduction.

Line 116 - it is true the ITS regions are too variable for designing universal primers, but they are flanked by conserved regions, and to the best of my knowledge ITS2 is not as variable in length as ITS1. So, instead of describing the variability of ITS regions as impeditive to short-amplicon primers design, I would rather discuss the fact that it cannot be aligned among unrelated taxa, and are not suitable for deeper phylogenies. Besides, it can only be used for taxonomical assignment if a somehow related group is represented in the database.

Line 133 - I would add consensus sequences 'from single individuals'. I was confused at first thinking that Nanopore could maybe do some sort of 'circular consensus', but if the consensus sequences are produced by homologous sequences from a single individual this should be made clear.

Line 141 - I would rephrase 'universal eukaryote'. Even though the primers could potentially work for all eukaryotes, there was no representative collection tested, and the authors stated themselves that there was a focus in animals.

Data Description and Analyses

Line 201 - following the idea above of exploring the universality of the primers, I would like to see some sort of figure or graph showing the representativeness of the different groups of eukaryotes in the 1000 sequences used for the primers design.

Line 214 - How was the quantification on an agarose gel performed? I would suggest a description how this was done and an evaluation of the pooling method in the Results/Discussion as fluctuations on samples sequence numbers may highly influence the efficiency and costs of the method.

Line 221 - Please inform the concentration of AMPure beads utilized

Results

Line 383 and Fig.2 - the authors state that at a distance of 4, samples had an increase in wrongly assigned sequences and a significantly lower accuracy in the consensus generated. However, what is shown in Fig. 2 is a box plot of pairwise distances of Nanopore sequences assigned to the sample against the Illumina consensus. How do the authors know that the sequences were wrongly assigned? Could they be assigned to other samples based on sequence distance? Is there a real change in the consensus sequence generated by the sequences assigned to a sample at a distance of 4? If so, why is that? Due to more indels and/or more mismatches? What are the features of the newly assigned sequences that

decrease the accuracy of the consensus? Could the higher distance at the barcode also incorporate sequences with more errors (i.e. is the number of errors in barcodes correlated to lower quality/more errors)? Are the errors distributed throughout the sequences? In my view it's important to understand the causes of lower accuracy, because absolute numbers, such as 2, 3 or 4 mismatches, might not represent the same issues when different barcode length, sequences or combinations are used.

Line 420 - please show examples of alignments with errors clustered in indel regions in the supplementary material. It is important for the reader to understand the patterns of errors found.

Line 429 - what could be the reason for a decrease in accuracy in higher coverages? Is this increase stochastic and no significant? Or is the incorporation of sequences with more (and maybe slightly repetitive) errors causing differences in the consensus? It would be very interesting to understand if the consensus creation is very sensitive to accumulation of identical errors, even if in small rates.

Lines 431 to 449 and Suppl. Figures 3 and 4 - even though I understand the value of presenting and summarizing results, the authors should not treat the data as representative neither for animals nor for plants. Please refer to the main groups analyzed (Arachnids, Insects and Magnoliopsida) and if there is an interest to compare to animals and plants in general, pick representative sequences from both groups (animals and plants) from public databases and present a comparison. For the data presented here, my suggestion would be one single boxplot graph for length difference presenting Arachnids, Insects and if wanted Magnoliopsida including both lengths excluding and including ITS regions, for a better understanding of the differences between full versus coding-only lengths. Another graph (or figure number) can summarize the same way the GC content.

Figures 3, 4 and 5 are presented before they are mentioned in the text.

Lines 471 to 483 and figure 3 - I wonder here what the value is in building such a phylogenetic tree including non-representative but yet arbitrarily picked species from three different kingdoms. Is the intent to show that the sequences produced by Nanopore are as accurate as sequences produced by other technologies and that differences/errors do not affect phylogenetic reconstruction? In that case, I would compare the tree with the data from same/similar species from databases for each group. Or is the intent showing that rDNA can be used to reconstruct true phylogenies for the groups? This doesn't seem to be part of the goals, but would demand other tests, again including many more sequences from databases. If the authors are presenting novelties and would like to place some group phylogenetically, there is nothing wrong (or better, it's the right thing to go) in picking representative sequences from databases for showing the correct phylogenetic placement of a group.

Lines 485 to 499 - Spiders are surely much better represented than other groups. But if there are other sequences in databases not represented here, I would include them. The question always goes back to the intent. Is it to show that the authors are contributing with valuable and correct rDNA sequences to populate databases? Then the improvement in phylogeny reconstruction should involve all sequences available and for which taxonomy can be trusted (I'm accounting here for possible errors or uncertainties in databases). This would reinforce the value of the approach in creating new references for an important and informative marker (or better saying, cluster of markers with different levels of divergence among different taxonomical groups), the rDNA region.

Line 531 and 532, Fig.6 and Discussion - the overlap between inter and intra-specific distance in rDNA might seem small but could have serious consequences if not interpreted well. Please show if in the dataset the distance would be impeditive of taxonomical assignment based on distance for some

lineages. One simple way would be to highlight the circles (e.g. make them darker) in Fig. 6 and suppl. Fig 5 with high intra-specific variability in rDNA for both intra and inter-specific distances. If the highlighted circles have high distance for inter-specific comparisons as well, this would not affect taxonomical classification, at least for those set of species/specimens presented. I wonder if the inter X intra-specific distances gap in COI is a natural one, or if in some cases COI was itself used for re-defining species boundaries, which would make the analysis redundant. In any case, it seems that in general it could be a good approach, even if it increases the level of Complexity, to sequence both rDNA and COI, as mentioned in the Discussion. I just wonder that, if COI could also be sequenced in the field with a similar approach (i.e. using Nanopore), as some samples might never reach the lab in the original country where the research is being conducted.
Lines 569-586 - The Illumina metabarcoding seem highly accurate when compared to Nanopore in Suppl. Fig 8 (please correct in line 574 the figure number), but this does not confirm that Illumina is quantitatively accurate, as the long amplicons generated huge biases. In fact, some taxa seem to have more than 2-fold difference in the Illumina results compared to their original frequency in the mock community based on Fig. 7. Was the adjustment per taxon performed as in reference [34]?
Discussion
Line 616 - I would like to see a bit deeper discussion on the feasibility for developing universal primers for sequencing full or partial mitogenomes across taxa, especially considering gene synteny and content within eukaryotes, apart from nucleotide variation.
Lines 625-626 - please rephrase to something similar to: long rDNA amplicons can potentially be amplified across diverse eukaryote taxa, here largely demonstrated in arthropods and arachnids, and in very small scale in fungi and plants.
Line 654 - rDNA is not only diploid but present in multiple (and unknown) number of copies, that might not be identical
Line 661 - it is not clear that even longer tails would not affect PCR, please either add a reference to confirm the statement or change it.
Line 666 - reference number is wrong, maybe [47]?
Nanopore metabarcoding - I think explanations are quite reasonable for justifying why certain taxonomical groups, especially those with shorter rDNA regions, would be preferentially amplified. However, given that the whole study focused on spiders, could the conditions be better optimized for spiders rather than other arthropods?
References
[22] is now published in Mol Ecol Resources

Methods

Are the methods appropriate to the aims of the study, are they well described, and are necessary controls included? Choose an item.

Conclusions

Are the conclusions adequately supported by the data shown? Choose an item.

Reporting Standards

Does the manuscript adhere to the journal's guidelines on minimum standards of reporting? Choose an item.

Choose an item.

Statistics

Are you able to assess all statistics in the manuscript, including the appropriateness of statistical tests used? Choose an item.

Quality of Written English

Please indicate the quality of language in the manuscript: Choose an item.

Declaration of Competing Interests

Please complete a declaration of competing interests, considering the following questions:

- Have you in the past five years received reimbursements, fees, funding, or salary from an organisation that may in any way gain or lose financially from the publication of this manuscript, either now or in the future?
- Do you hold any stocks or shares in an organisation that may in any way gain or lose financially from the publication of this manuscript, either now or in the future?
- Do you hold or are you currently applying for any patents relating to the content of the manuscript?
- Have you received reimbursements, fees, funding, or salary from an organization that holds or has applied for patents relating to the content of the manuscript?
- Do you have any other financial competing interests?
- Do you have any non-financial competing interests in relation to this paper?

If you can answer no to all of the above, write 'I declare that I have no competing interests' below. If your reply is yes to any, please give details below.

I declare that I have no competing interests

I agree to the open peer review policy of the journal. I understand that my name will be included on my report to the authors and, if the manuscript is accepted for publication, my named report including any attachments I upload will be posted on the website along with the authors' responses. I agree for my report to be made available under an Open Access Creative Commons CC-BY license (http://creativecommons.org/licenses/by/4.0/). I understand that any comments which I do not wish to be included in my named report can be included as confidential comments to the editors, which will not be published.

I agree to the open peer review policy of the journal

To further support our reviewers, we have joined with Publons, where you can gain additional credit to further highlight your hard work (see: https://publons.com/journal/530/gigascience). On publication of this paper, your review will be automatically added to Publons, you can then choose whether or not to claim your Publons credit. I understand this statement.

Yes Choose an item.
